# Head posture mediates the association of cognition with hand grip and pinch strength in older adults: an examination using structural equation modeling

**DOI:** 10.1186/s12891-023-06436-4

**Published:** 2023-04-25

**Authors:** Halil Ibrahim Celik, Banu Unver, Eda Akbas, Emin Ulas Erdem

**Affiliations:** 1 Department of Physiotherapy and Rehabilitation, Faculty of Health Sciences, Lokman Hekim University, Ankara, Turkey; 2 Bilge Çocuk Special Education and Rehabilitation Center, Ankara, Turkey; 3 Department of Physiotherapy and Rehabilitation, Faculty of Health Sciences, Bulent Ecevit University, Zonguldak, Turkey

**Keywords:** Older, Cognition, Forward head posture, Pinch strength, Grip strength

## Abstract

**Background:**

The association of cognition with hand grip and pinch strength has been well-recognized in older adults. The authors sought to explore: (1) associations among forward head posture (FHP), cognition, and hand grip and pinch strength in older adults; and (2) the mediator role of FHP in these pathways using structural equation modeling (SEM).

**Methods:**

This cross-sectional study included 88 older adults (70.5% male; mean age = 68.75±3.87 years). Cognition was assessed by the Mini-Mental State Examination (MMSE), head posture by the Craniovertebral Angle (CVA) obtained from photographic analysis, hand grip strength by a handheld dynamometer, and pinch strength by a pinch meter. Using the two SEMs, a potential mediator role of the CVA was investigated. While the MMSE was addressed as an independent variable in both models, hand grip and pinch strength were addressed as dependent variables in model 1 and model 2, respectively.

**Results:**

The correlations between the CVA and MMSE (r = 0.310), hand grip strength (r = 0.370), and pinch strength (r = 0.274 to 0.292) were statistically significant (p < 0.001). In addition, significant associations were found between the MMSE and hand grip and pinch strength, ranging from 0.307 to 0.380 (p < 0.001). The mediation analysis showed that the standardized total (β = 0.41, p < 0.001) and indirect (mediated) effects (β = 0.12, p = 0.008) of the MMSE on hand grip strength were significant in model (1) The results were similar for model (2) The standardized total (β = 0.39, p = 0.001) and indirect effects (β = 0.10, p = 0.026) of the MMSE on pinch strength were significant. As a partial mediator in both models, the CVA explained 29% and 26% of the total effect in models 1 and 2, respectively.

**Conclusions:**

The CVA was associated with the MMSE, hand grip strength, and pinch strength, and CVA partially mediates the association of the MMSE with grip and pinch strength in older adults, indicating that cognition had an effect on grip and pinch strength through an indirect path via head posture. This finding reveals that evaluating head posture and providing corrective therapeutic interventions as needed may be beneficial in reducing the negative impact of decreased cognition on motor functions in older adults.

## Introduction

Hand grip strength has been suggested as a biomarker of ageing. It has been associated with overall strength, upper limb function, falls, quality of life, disability, morbidity, and even mortality in older adults [1]. Hand grip strength has also been associated with cognition and this association has been well-documented in older adults. Cui et al. reported in a recent meta-analysis that poorer grip strength was associated with cognitive decline and that grip strength may be an early indirect marker of subsequent cognitive decline [2]. In a systematic review, Kobayashi-Cuya et al. reported that hand grip strength was associated with cognition cross-sectionally and longitudinally in older adults and that variables such as socio-demographics, depression, physical activity level, and medical conditions may affect this association [3]. On the other hand, although studies on pinch grip are less common, it is known to be essential for performing the majority of daily living activities such as buttoning a shirt, closing a jacket’s zipper, unlocking doors, retrieving coins from a purse, or writing a short note. An age-related decline in pinch strength is associated with a reduction in daily living activity performance, which may have an effect on quality of life in older adults [4, 5]. In addition, studies have shown that pinch strength is also associated with cognition in older adults, like hand grip strength [4, 6, 7] and that variables such as socio-demographics and medical conditions (e.g. stroke, arthritis) may affect this association [8, 9].

Forward head posture (FHP) is one of the most common postural deviations related to advanced age in older adults and is defined as the excessive anterior positioning of the head in the sagittal plane relative to the shoulder [10]. To our knowledge, there is no study examining the association of FHP with cognition, hand grip strength, and pinch strength, and the effect (mediator role) of FHP on the association of cognition with hand grip and pinch strength in older adults. Therefore, the aims of the current study were to explore: (1) associations among FHP, cognition, hand grip strength, and pinch strength in older adults; and (2) whether FHP serves as a mediator in these pathways. We hypothesized that FHP would be associated with other variables and that older adults with higher cognitive function would have better head posture, and in turn, greater hand grip and pinch strength.

## Methods

This cross-sectional study was carried out at Zonguldak Bülent Ecevit University Department of Physiotherapy and Rehabilitation between December 2020 and July 2021. The study was approved by the ethics committee of the university and conducted in accordance with the Helsinki Declaration. Prior to recruitment in the study, all participants were informed about the objective and scope of the study, and they signed an informed consent form.

### Participants

The study was conducted with a convenience sample of 88 community-dwelling older adults aged 65 years or older. They were recruited from the University Hospital in Turkey. They were excluded if they had radiculopathy; a history of cervical spine or upper limb surgery; neurological diseases; communication deficits (inability to communicate verbally or in writing); or, arthritis or pain in the hand/wrist/arm. All participants were able to cooperate with instructions and assessments.

### Procedure

After the demographic data of the participants were recorded, cognition was assessed using the Mini-Mental State Examination (MMSE) and forward head posture (FHP) was assessed using the photographic analysis. Finally, hand grip strength and pinch strength were evaluated using a Jamar hand dynamometer (Lafayette Instrument Company, USA) and a Baseline Hydraulic Pinch Gauge (Fabriction Enterprises, Inc.), respectively.

### Outcome measurements

The MMSE was used to assess global cognitive function, which consists of 11 items that evaluate attention and orientation, memory, registration, recall, calculation, language, and ability to draw a complex polygon. Higher MMSE scores show improved cognitive functioning. The maximum total score is thirty [11].

FHP was evaluated by measuring the craniovertebral angle (CVA) using a digitized, lateral-view photograph of the participant in a usual standing posture [12]. Using a digital camera that was positioned 1.5 m away on a stable base and at shoulder level, all photographs were taken from the dominant side. The tragus of the ear was marked and a plastic marker was inserted over the seventh cervical spinous process. After taking the photograph, using the ImageJ software (version 1.52 k; National Institutes of Health, Bethesda, MD, USA), the CVA (°) was calculated as the angle between the horizontal line passing through the C7 spinous process and the line connecting the seventh cervical vertebra and the tragus of the ear [13]. Lower CVAs indicate increased FHP. The CVA measurement with photographic analysis has demonstrated high reliability (ICC = 0.98) [14].

A Jamar handheld dynamometer (Lafayette Instrument Company, USA) was used to measure hand grip strength and a Baseline Hydraulic Pinch Gauge (Fabriction Enterprises, Inc.) was used to measure strength of the various pinch types. The strength of three pinch types were measured: lateral pinch (the thumb pad to the lateral aspect of the middle phalanx of the index finger, also known as key pinch), three-point pinch (between the thumb, index, and middle fingers, also known as three jaw chuck pinch), and tip pinch (thumb tip to the index tip, also known as two-point pinch) [15]. According to the protocol established by the American Society of Hand Therapists, participants were seated on a standard-height chair without armrests with the shoulder adducted and neutrally rotated, elbow flexed at 90°, and the forearm and wrist neutrally positioned. Because previous research has found an association between the ability to perform activities of daily living and pinch strength for the dominant hand only, the measurements were taken with the dominant hand only [15]. Three consecutive measurements with a one-minute interval between the measurements were performed to evaluate hand grip and pinch strength with the participant exerting maximum force to grip or pinch with verbal encouragement. Analysis was conducted using the average value obtained from all three trials [16].

### Statistical analysis

Statistical Package for the Social Sciences 26.0 (SPSS Inc., Chicago, USA) was used for descriptive and univariate analyses of the data, and the Analysis of Moment Structures (AMOS) Graphics 23.0 (Small Waters Corp., Chicago, USA) was used for performing the structural equation modeling (SEM), obtaining maximum-likelihood estimates of model parameters, and providing goodness-of-fit indices. The univariate normal distribution of the data was examined using visual (histogram and probability graphs) and analytical methods (KS-SW tests). These tests showed that all numerical variables fitted to a normal distribution. Therefore, the Pearson product-moment correlation test was used to assess bivariate associations. Mardia’s multivariate kurtosis coefficient was used to evaluate whether the data exhibit a multivariate normal distribution, which was found to be less than 5, that is acceptable (for model 1 and 2, it was 2.36 and 1.23, respectively) [17].

Two SEMs with FHP in the mediator role were proposed. While cognition was addressed as an independent variable in both models, hand grip strength and pinch strength were addressed as dependent variables in model 1 and model 2, respectively. The only latent variable included in the models was pinch strength in model 2, which was represented by three observed variables (lateral, three-point, and two-point pinch strength). Since the loadings for the pinch strength were adequate (i.e., λ’s > 0.40, see Fig.  2), these observed variables were maintained in model 2 [18]. The magnitude of χ2 divided by its degrees of freedom (χ2/DF: values five or less), Root Mean Square Error of Approximation (RMSEA: <0.08 acceptable and < 0.05 excellent), Comparative Fit Index (CFI), Goodness of Fit Index (GFI), Tucker Lewis index (TLI), and Normed Fit Index (NFI) (for CFI, GFI, TLI, and NFI: >0.90 acceptable and > 0.95 excellent) were used as indices to assess the models’ fit [19]. In all cases, a p-value of 0.05 or less was selected as an indication of statistical significance for univariate and multivariate tests.

**Fig. 1:**
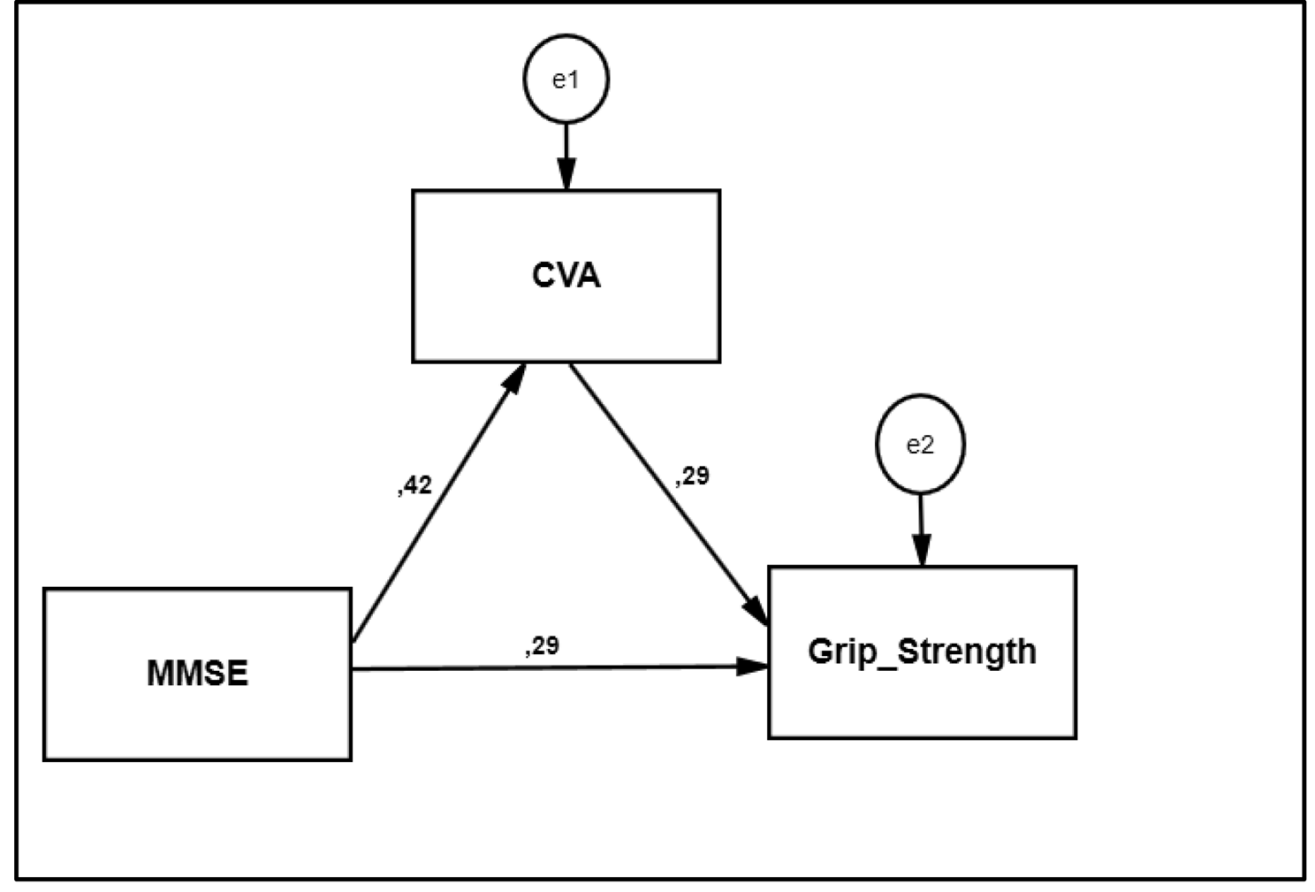
The model 1 illustrating the mediating roles of CVA in the association between MMSE and grip strength. Notes: Standardized regression weights are shown for the associations between each variable

The sample size for the SEM is generally recommended to be at least 5 cases per free parameters in the model to preserve statistical stability. The minimum sample required for this study was calculated as 40 and 85 since model 1 and 2 had 8 and 17 free parameters, respectively. Hence, our sample of 88 older adults was sufficient to conduct SEM [20].

## Results

The demographic features, MMSE scores, CVA values, and grip and pinch strength of the participants are presented in Table 1. This study included 88 (62 male, 26 female) older adults with an average age of 68.75±3.87 years. Table 2 shows the bivariate associations between the variables included in the SEM models. The correlations between the CVA and MMSE (r = 0.310), hand grip strength (r = 0.370), and pinch strength (r = 0.274 to 0.292) were statistically significant (p < 0.001). In addition, significant associations were found between the MMSE and hand grip and pinch strength, ranging from 0.307 to 0.380 (p < 0.001).

**Table 1:** Demographic and clinical characteristics of the participants (n = 88)

Parameters	Mean ± SD	Range
**Age (y)**	68.75 ± 3.87	65.0 to 84.0
**Years of education**	5.38 ± 2.87	0 to 16
**Height (cm)**	165.19 ± 7.60	145.0 to 180.0
**Weight (kg)**	78.01 ± 14.27	53.0 to 125.0
**BMI**	28.72 ± 5.69	19.03 to 46.09
**MMSE**	23.64 ± 3.99	14.0 to 30.0
**CVA (°)**	35.01 ± 8.84	16.25 to 53.13
**Grip strength (kg)**	27.90 ± 8.90	7.33 to 47.33
**Lateral pinch strength (kg)**	4.06 ± 2.41	10
**Three point pinch strength (kg)**	2.86 ± 2.01	7.33
**Two point pinch strength (kg)**	1.90 ± 1.72	8.0

BMI, Body Mass Index; CVA, craniovertebral angle; MMSE, Mini-Mental State Examination

**Table 2:** Correlation matrix of variables in SEM models

Parameters	MMSE	CVA (°)	Grip strength (kg)	Lateral pinch strength (kg)	Three point pinch strength (kg)	Two point pinch strength (kg)
**MMSE**	1.00					
**CVA (°)**	0.310^*^	1.00				
**Grip strength (kg)**	0.380^*^	0.370^*^	1.00			
**Lateral pinch strength (kg)**	0.307^*^	0.292^*^	0.775^*^	1.00		
**Three point pinch strength (kg)**	0.346^*^	0.290^*^	0.743^*^	0.840^*^	1.00	
**Two point pinch strength (kg)**	0.342^*^	0.274^*^	0.631^*^	0.699^*^	0.769^*^	1.00

Pearson Product-Moment Correlation Test. *p < 0.01; MMSE, Mini-Mental State Examination; CVA, craniovertebral angle

**Fig. 2:**
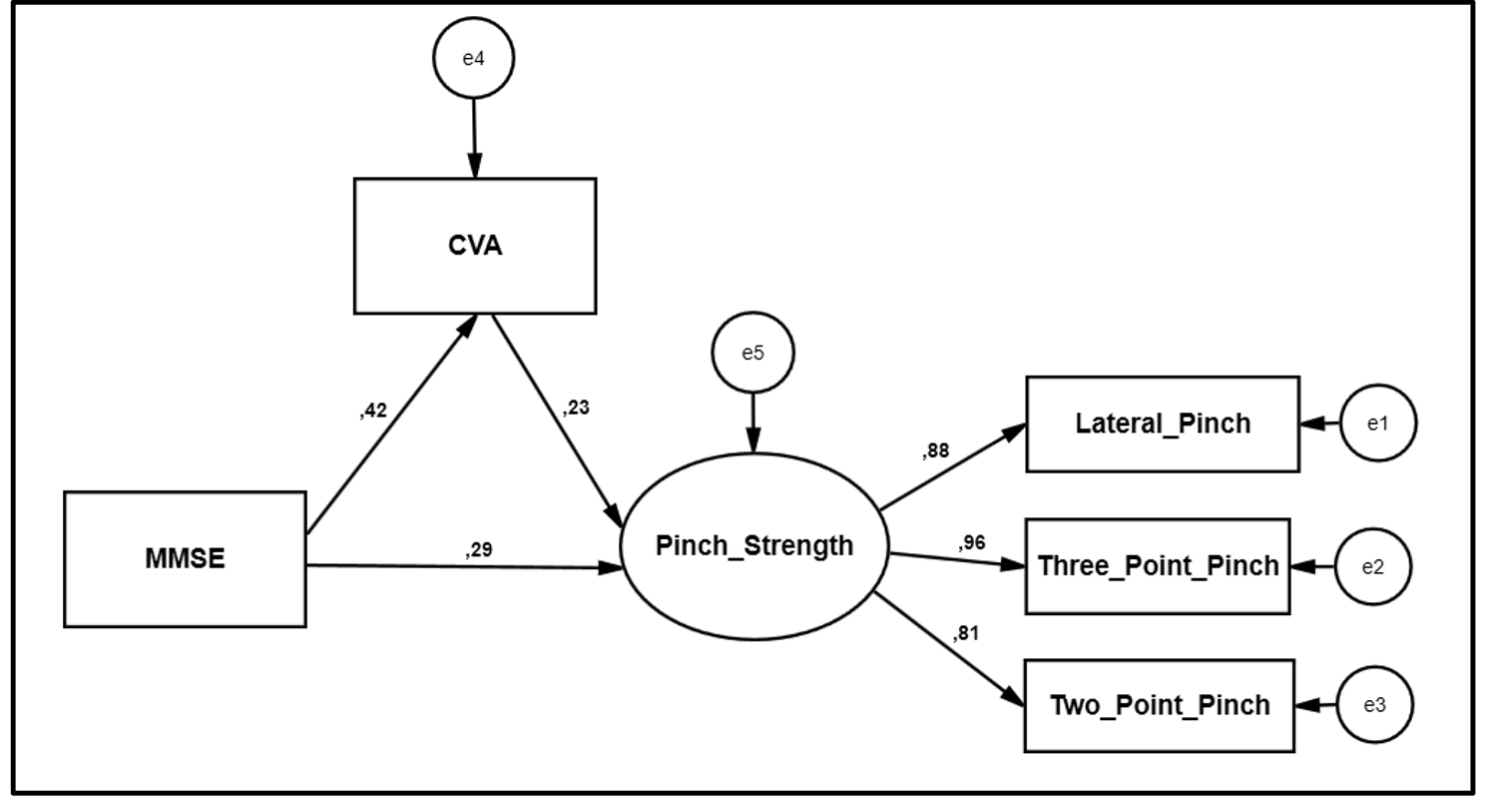
The model 2 illustrating the mediating roles of CVA in the association between MMSE and pinch strength. Notes: Standardized regression weights are shown for the associations between each variable

The proposed models 1 and 2 are shown in Figs. 1 and 2. The values shown next to the single-headed arrows were the estimated standardized regression coefficients. All coefficients were statistically significant (p < 0.05). Both model demonstrated good to excellent fit to the data, with the exception of the RMSEA value for model 1 (Table 3). However, it was reported that RMSEA may incorrectly indicate a poor fit, especially in simpler SEM with relatively few degrees of freedom [21]. Therefore, based on other fit indices, it was concluded that the fit of the model 1 is adequate.

**Table 3:** The goodness of fit indices for model 1 and 2

	χ^2^	DF	χ^2^/ DF	CFI	GFI	TLI	NFI	RMSEA
**Model 1**	1.89	1	1.89	0.97	0.99	0.90	0.94	0.10
**Model 2**	3.01	5	0.60	1.00	0.99	1.00	0.97	< 0.001

χ^2^, Chi-Square; DF, Degrees of Freedom; CFI, Comparative Fit Index; GFI, Goodness of Fit Index; TLI, Tucker Lewis Index; NFI, Normed Fit Index; RMSEA, Root Mean Squared Error of Approximation

The association between cognition and forward head posture and the association between forward head posture and hand grip strength were examined. Consistent with a mediation model, the MMSE had a significant effect on the CVA (β = 0.42; *P* < 0.001). The CVA, in turn, had a significant effect on the hand grip strength (β = 0.29; *P* = 0.009) (Table 4). Afterwards, the standardized total effects, indirect effects (mediated by forward head posture), and direct effects (independent of forward head posture) of cognition on hand grip strength were examined. The total effect of the MMSE on hand grip strength was β = 0.41 (*p* < 0.001). The indirect effect was β = 0.12 (*p* = 0.008), and the direct effect was β = 0.29 (*p* < 0.001), indicating that the CVA, as a mediator, accounted for 29% of the association between the MMSE and hand grip strength (Table 4).

**Table 4:** Path coefficients of the hypothesized model

Association among MMSE, CVA, and hand grip strength			β	95% CI	p
MMSE → CVA			0.42	0.37 to 0.48	**< 0.001**
CVA → Hand Grip Strength			0.29	0.12 to 0.44	**0.009**
MMSE → CVA → Hand Grip Strength					
	Direct effects		0.29	0.15 to 0.48	**< 0.001**
	Indirect effects		0.12	0.05 to 0.19	**0.008**
	Total effects		0.41	0.29 to 0.53	**< 0.001**
% Total effects mediated by CVA: **29%**					
**Association among MMSE, CVA, and precision grip strength**			**β**	**95% CI**	**p**
MMSE → CVA			0.42	0.37 to 0.48	**< 0.001**
CVA → Precision Grip Strength			0.23	0.06 to 0.38	**0.028**
MMSE → CVA → Precision Grip Strength					
		Direct effects	0.29	0.14 to 0.44	**0.003**
		Indirect effects	0.10	0.03 to 0.16	**0.026**
		Total effects	0.39	0.26 to 0.52	**0.001**
% Total effects mediated by CVA: **26%**					

Note: Standardized coefficients reported. Bootstrap sample = 5,000 with replacement. MMSE, Mini-Mental State Examination; CVA, craniovertebral angle

The results were similar for the model 2, in which the CVA mediated the association between the MMSE and pinch strength. The standardized total effects, indirect effects, and direct effects of the MMSE on pinch strength were β = 0.39 (*p* = 0.001), β = 0.10 (*p* = 0.026), and β = 0.29 (*p* = 0.003), respectively. The indirect effect of the CVA accounted for 26% of the total effect (Table 4).

## Discussion

To our knowledge, there is no study examining the association of FHP with cognition, hand grip strength, and pinch strength, and the mediator role of FHP on the association of cognition with hand grip and pinch strength in older adults. Considering that FHP is one of the most common postural deviations related to advanced age in older adults, this is remarkable. Hence, the current study is the first to reveal that FHP was associated with cognition, hand grip strength, and pinch strength, and FHP serves as a mediator in these pathways in older adults. The study’s findings support the hypothesis that “higher cognitive function would be associated with better head posture, and better head posture, in turn, would be associated with greater hand grip and pinch strength.”

### Association of cognition with the hand grip and pinch strength

Although research shows a positive association between cognition and motor function such as hand grip and pinch strength in older adults, the direction of causality of the association are uncertain and still under discussion. Most explanations for this association fall into one of three categories: motor function directly affects cognition; cognition directly affects motor function; a third factor affects both functions simultaneously [22]. In addition, the underlying mechanism of this association is also not fully understood. While some authors speculate that this association may depend on the fact that cognitive and motor areas of the central nervous system share common neural processes [23], some authors speculate that various processes, including alternations in the white matter, telomere shortening, white matter integrity, and processes operating at the cellular level may operate together [22, 24]. In the current study, we focused not on understanding the direction of causality and the underlying mechanism of the association but on a hypothesis suggesting head posture mediates the association between cognition and motor function in older adults. Hand grip and pinch strength, which can be considered as the components of motor function in the current study, were associated with cognition. These findings are consistent with earlier findings reporting the positive association of cognition with the hand grip and pinch strength in older adults [2, 3, 8].

### Association between cognition and FHP

Although little research has been conducted, some evidence has emerged regarding the association between cognition and head posture in older adults similar to the current study, and it has been proposed that this association is attributed to dopamine, which is known to be crucial for postural alignment and cognition [25].

### Association of FHP with hand grip and pinch strength

The current results show that FHP was associated with reduced hand grip and pinch strength in older adults. Some studies have speculated that head/neck position may affect grip strength in young adults, but these studies have not addressed FHP, a structural postural deviation [26–28]. Although the association mechanism of head posture/position with grip strength is unclear, some explanations have been suggested. First, it can be explained by Janda’s theory, which postulates that the upper extremity is a chain of motion that begins with the cervical and thoracic spine and ends with the fingers and that any changes within this chain may be the result in poor grip performance [29]. Another possible explanation is that FHP adversely affects proximal stabilization essential for distal movement by compromising scapular alignment and kinematics, which may indirectly affect grip strength [30]. This explanation was supported by many previous studies which have demonstrated that FHP changes scapular kinematics because the axioscapular muscles that attach the spinal column with the scapula, such as the trapezius, serratus anterior, and scapula elevators, are affected by FHP [31–33].

The results of studies on young adults investigating how head/neck position affects hand grip strength have been inconclusive. Some studies have found that head/neck position had no effect on hand grip strength [26, 34]. On the contrary, it was has also been reported that the hand grip strength was significantly higher in the neutral and rotational positions of the head/neck compared to other positions [27, 28]. These inconclusive results may be attributable to the fact that these studies have focused on the effect of head/neck position, not the structural postural deviation of the head, on hand grip strength and because they have included asymptomatic young adults. In the only study that examined the effect of FHP on hand grip strength, Mosaad et al. reported no difference in hand grip strength between young adults with and without FHP [35]. This may arise from the small mean difference (~ 8^0^) in the CVA between groups with and without FHP, that is, the severity of the head postural deviation was insufficient to cause distal alternations in the upper extremity.

### FHP as a mediator of the association of cognition with hand grip and pinch strength

Although there are studies that address several mediator factors such as socio-demographics, depression, physical activity level, and medical conditions in the association of cognition with hand grip and pinch strength in older adults [3, 4, 6, 7], the current study is the first to address FHP as a mediator factor in this association. Our results revealed a novel contribution that FHP partially mediates this association in older adults, demonstrating how cognition indirectly affect hand grip and pinch strength through head posture.

The current study had several limitations. First, we investigated only cross-sectional associations, which cannot describe the causal direction of effects between variables. Yet, the current study can provide preliminary data for further longitudinal or experimental studies that can verify the findings of the study. Second, the use of convenience sample of predominantly male participants may limit the generalizability of the findings. Future studies should include more homogeneous sample with a gender balance. Third, the MMSE, a global assessment tool, was used to evaluate cognition. The association of different cognitive domains with head posture and hand grip and pinch strength needs to be investigated further.

## Conclusions

The CVA was associated with the MMSE, hand grip strength, and pinch strength, and CVA partially mediates the association of the MMSE with grip and pinch strength in older adults, indicating that cognition had an effect on grip and pinch strength through an indirect path via head posture. The research findings reveal that evaluating and enhancing cognition and head posture may help to improve hand grip and pinch strength. Furthermore, emerging evidence suggests that evaluating head posture and providing corrective therapeutic interventions as needed may be beneficial in reducing the negative impact of decreased cognition on hand grip and pinch strength in older adults.

## Data Availability

The data that support the findings of this study are available from the corresponding author upon reasonable request.
